# Observations on Lock Jaw, and Tetanus, with Cases

**Published:** 1809-05

**Authors:** John Howship

**Affiliations:** Member of the Royal College of Surgeons in London, and Assistant Surgeon to the 82d Regiment of Foot


*Observations on Lock Jaw, and Tetanus, with Cases. By* John Howship, Member of the Royal College of Surgeons in London, and Assistant Surgeon to the 82d Regiment of Foot.

WHILE our knowledge relating to the true manner in which the healthy actions and functions of the brain, and nervous system, are carried on, continues to be so very limited as at present, it need excite little surprise if, while attending to the phenomena of nervous diseases, we sometimes meet with circumstances that naturally tend to set these complaints in a new point of view.

In the particular description of spasm constituting tetanus, the relation existing between the local and general affection has sometimes been rather misunderstood. Lock jaw has been allowed to be of the same nature with tetanus, nus, but, in some cases, it has been said that the former affection may arise and continue without inducing the latter, although in most instances they both appear in the same individual.

From attentively reflecting upon these diseases, however, it seems to be more consistent with truth, to consider that a lock-jaw naturally passes, and will very constantly pass on to tetanus; and for why? Because the disease depends on a morbid state of the whole nervous system, or, according to Dr. Crichton's theory of the nervous influence, on an altered state of the sentient principle secreted into the nerves.

This morbid condition of the nerves very probably exists in the previous state of the constitution, as the predisponent cause of the disease; and would more generally, but sometimes local or general derangement of the vascular action will rouse into activity the secret energies of this destructive malady.

We know of no practice that can, with confidence, be recommended for arresting the progress of the peculiar diseased impressions, upon which the production of this disease depends. Much obscurity must be expected to dwell upon this subject, as the spasm in question is the consequence of an irritation, distant and distinct from the brain, and totally unconnected with local derangement in the head, or any of the phenomena of compression; by reason of which, the doctrines now so clearly understood, capable of guiding our practice successfully in most complaints arising from an oppressed state of the brain, fail us here altogether.

One point however seems clear; it is this, that the progress of the diseased impression is in the first instance very slow, in the generality of examples. It seems to creep, as it were, from the wound upwards to the origin of the nerve; from thence, extending its influence to the whole nervous fabric of the sensorium, emanating as from a centre, in the various course of the particular nerves distributed throughout the body and extremities.

This must not be understood as being the constant, although it is the very general manner in which the first circumstances attending this disease occur. The course of the morbid irritation acting upon the brain from the wound, producing no apparent disturbance upon the muscular parts in the neighbourhood, nor inducing any new or peculiar affection in the sensorium itself, the first intimation of the presence of this terrible disorder being the consequence of of the diseased impression being conveyed, by reflection, back from the brain upon those muscular structures with which the minute branches of the nervous fibres are interwoven.

The progress of the morbid impression from the wound towards the head is slow; from the brain also, it is presumed the irritation passes with a very slow movement, because the nerves supplying the muscles situated about the face, throat, and neck, are uniformly observed to give the earliest intimations of the disease; and it is according to the rapidity with which the tendency spreads, whether the same disposition manifests itself in the muscles surrounding the chest, loins, and limbs, in one, or in twenty-one days afterwards.

The above is considered to be by far the most general progress of the complaint; but in particular circumstances it has happened, that instead of the irritation only passing silently from the wound upwards to the head, it has produced a sudden and most painful spasm, affecting all the large muscles around which action has passed from the thigh downwards, and disappeared altogether by going out, according to the patient's own words "at the toe ends."

The peculiar and hidden derangement in the action or state of the nerves upon which the disease depends, being in all probability connected with change in structure; may leave a very reasonable doubt on the mind, as to the chance of a favourable result in an operation, where the situation of the wounded part would admit of its complete removal; particularly if the general diffusion or establishment of this irritation has already evinced itself by fits of tetanus.

On the other hand however it has happened, that a lock-jaw has been relieved by a removal of the limb in which the wound had been received, and the man recovered; the exciting cause of the disease having been removed before the reflex irritation had gone beyond the nerves of the jaws and face. Again, a complete example of lock-jaw, with successive and dreadfully violent fits of tetanus, a very tedious case, has ended fortunately, and that while the wounded toes, from which all the mischief sprung, was very little interfered with by art.

*Case of Tetanus illustrative of the peculiar sympathies existing in the nervous system under the influence of disease.*

M. Wheelan, of his Majesty's Ship Colossus, aged 18 years, was wounded in many parts of his body by splinters ters of various descriptions. A very large wound was behind the right shoulder, where the whole of the integuments were carried clear away, and the posterior mass of the deltoid, together with those other muscles in its immediate neighbourhood, were consequently exposed.

This unfortunate lad had several other large wounds, deep and foul, about the back; besides which, the sores of a smaller description were without number; exhibiting in the different appearance of each ulceration, all the gradations from the perfectly healthy, to the worst and most unpromising description of wound from gun-shot.

In the larger wounds, the sloughy membranous matter did not separate earlier than the eleventh day, at which time the smaller ulcers looked perfectly well, clean, and moistened by a bland, natural, purulent discharge.

On the evening of the eighth day, he complained of a pain about his face, as if his jaws were catching, or snatching together. The same uneasiness was felt between the glottis and the apex of the chin, from the affection spreading to the muscles of the tongue and larynx.

On the tenth day, the ung. hydrarg. was rubbed in, ʒi. every hour; while internally calomel, gr. ij. opii purif. gr. i. was ordered, and every second hour repeated. The same evening his mouth was affected. Two blisters were also applied over the masseter muscles.

While dressing the wounds upon the back, a very remarkable effect was observed. The more trifling sores were first covered with fresh ointment; and although every care was taken to lay the dressings on tenderly, yet they gave pain, of which frequent complaint was made. The two worst wounds in the loins were foul and deep, ragged, and unhealthy. In laying the yellow resin ointment in contact with the inside of these wounds, not the least sensation of pain was felt in or near the wound, yet several times the muscles of the jaws and breast were twitched in the same instant that the wounded surface was disturbed by the application of the dressings. This curious circumstance, on inquiry, the lad could not explain to me, as I explained it to myself. He was not conscious of the least connection between the one complaint and the other. He said he felt no pain near the wound, but that the knitting of his teeth, and the working of his breast, came on by accident. This is the sense of what he expressed, rather than the words he used; for persons afflicted in this most painful complaint, rarely go about to explain satisfactorily what they feel, to such as desire to learn; these must must be first discovered by suggestions, requiring only a token in answer, and then confirmed by cross questions, which will ascertain whether the patient has signified correctly or not.

This lad was of a costive habit of body, on which account it was frequently necessary to order him aperient medicines; this might, in some degree, have been the means of aggravating the violence of the symptoms. He often called for drink, which he seemed to take eagerly; but as often as he swallowed, a part of the fluid was forced back again out of his mouth by the force with which the chest was influenced by the convulsion; during these moments, the features of his face were very much disturbed, and the teeth fixed most firmly together by the increased violence of the spasm.

On being questioned, he said, that since he began spitting, he felt less pain in the parts about the shoulder than he did before.

This young man was frequently convulsed during the eleventh day and night. On the morning of the twelfth day, I observed that his sufferings were very great, with which he was extremely restless during the intervals. He with assistance got up, and sat on the side of the bed; and notwitstanding the attempt to swallow was attended with terrible convulsions, affecting the whole series of muscles subservient to respiration, still he succeeded in drinking some water.

His wounds were just about to be dressed, when Mr. Whyte, the surgeon of the Belerophon passed by, and observed, whether it might not be better to pass on to the rest for a time? About half an hour after this, when again settled in bed, he was suddenly seized with a convulsive attack, and for ten minutes his extremities were in a state of continued tremor, much like the cold fit of an intermittent; his eyes during this interval were fixed, and turned upwards. His respiration perfectly easy and natural; his breast heaved with the greatest apparent tranquillity, while the pulse was perfectly even, and not in the least degree hurried.

The tremors of the limbs gradually subsided, his eyes and eye-lids relaxed, and he slowly recovered his consciousness. Though well nigh exhausted by his sufferings, he now cast his eyes with languid expression upon those that stood around him. He had, however, scarcely time to recollect himself, when his breast began to heave laboriously, and his jaws became violently knit together. In the the last fit the system of voluntary muscles were convulsed. In the present attack, all the involuntary actions in the body gave way. The breast heaving with violent and frequent throes, gave the most impressive signs of approaching dissolution. Upon laying the hand upon the breast, the heart was felt struggling hard amid the surrounding destruction. The pulse was now very much hurried, and soon afterwards became unequal, and then intermittent. The breast threw up the air from the lungs by jerks, and the mouth foamed from the convulsive actions of breathing, as well as from the commencing salivation. Respiration was now only continued at intervals, and the heart consequently acted without its proper stimulus being conducted to it, therefore the pulse gave long intermissions.

A deep heave of the breast repeatedly set the heart into action, after it had threatened to cease. The eyes for some time remained lively, indicating the soul being still in possession of her proper seat; but when the action of the chest had nearly terminated, and the colour of the body was manifestly changing, the eyes became languid and fixed; and lastly, they bespoke death having closed the scene.

*Scarbro'*, *April* 18, 1809.

(To be continued.)

